# *Helicobacter pylori* Pathogen-Associated Molecular Patterns: Friends or Foes?

**DOI:** 10.3390/ijms23073531

**Published:** 2022-03-24

**Authors:** Daniela Eletto, Fatima Mentucci, Antonia Voli, Antonello Petrella, Amalia Porta, Alessandra Tosco

**Affiliations:** 1 Department of Pharmacy, University of Salerno, 84084 Fisciano, Italy; fmentucci@unisa.it (F.M.); avoli@unisa.it (A.V.); apetrella@unisa.it (A.P.); aporta@unisa.it (A.P.); 2 Ph.D. Program in Drug Discovery and Development, University of Salerno, 84084 Fisciano, Italy

**Keywords:** PAMP, LPS, ADP-heptose, OMV

## Abstract

Microbial infections are sensed by the host immune system by recognizing signature molecules called Pathogen-Associated Molecular Patterns—PAMPs. The binding of these biomolecules to innate immune receptors, called Pattern Recognition Receptors (PRRs), alerts the host cell, activating microbicidal and pro-inflammatory responses. The outcome of the inflammatory cascade depends on the subtle balance between the bacterial burn and the host immune response. The role of PRRs is to promote the clearance of the pathogen and to limit the infection by bumping inflammatory response. However, many bacteria, including *Helicobacter pylori*, evolved to escape PRRs’ recognition through different camouflages in their molecular pattern. This review examines all the different types of *H. pylori* PAMPs, their roles during the infection, and the mechanisms they evolved to escape the host recognition.

## 1. Introduction

In 1989, Janeway proposed the pattern recognition theory defining PAMPs (Pathogen-Associated Molecular Patterns), the microbial components not found in eukaryotic hosts recognized as non-self by several innate immune receptors (Pattern Recognition Receptors: PRRs) during an infection. The host cell, warned of the presence of potentially dangerous microorganisms by the PAMPs, responds by producing inflammatory mediators and recruiting immune cells to fight infection [1].

This conceptual framework for understanding innate immune recognition was later validated experimentally and has become a paradigm [2]. To be recognized by the innate immune system, PAMPs must have three main features. First, within a given class of microorganisms, they should be invariant, so that they can be detected by a limited number of germ-line encoded receptors. Second, they should only be present in microorganisms and not in eukaryotic cells in order to allow for discrimination between self and non-self. Third, they should have essential roles in microbial physiology, so that their detection cannot be easily eliminated via mutation. Indeed, bacterial PAMPs are often cell wall constituents, such as lipopolysaccharide, peptidoglycan, lipoteichoic acids, and cell wall lipoproteins.

Examples of PAMPs of particular importance include lipid A, which is a constituent of the Gram-negative lipopolysaccharide (LPS), recognized by Toll-like receptor 4 (TLR4); flagellin, a protein that polymerizes to form the flagellum, sensed by TLR5; lipoproteins from both Gram-positive and Gram-negative bacteria, recognized by TLR2; bacterial DNA containing CpG motifs that stimulate TLR9; and fragments of bacterial peptidoglycan that are detected in the host cell cytosol by NOD1 and NOD2 (Nucleotide-binding Oligomerization Domain-containing 1 and 2) receptors.

*Helicobacter pylori* is a highly successful human pathogen infecting approximately 50% of the world’s population [3]. It can successfully colonize the human stomach for a long time, escaping the host immune system and causing a prolonged infection with subsequent chronic gastritis, one of the most common risk factors for gastric malignancy. The pathological outcomes of *H. pylori* chronic colonization depend on the host–pathogen interactions, which are heavily influenced by bacterial and host genetics, and environmental factors.

**Figure 1:**
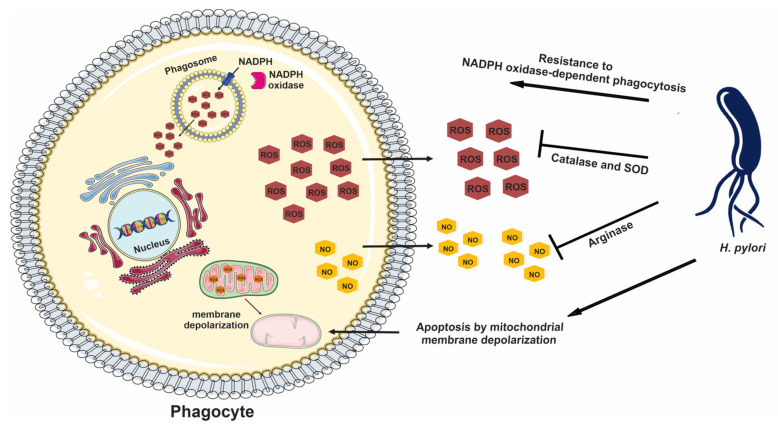
*H. pylori* survival strategies against phagocytes. The bacterium secretes catalase and SOD to reduce ROS toxicity, synthetizes arginase to limit NO production, reduces host NADPH oxidase response, and induces macrophage apoptosis.

The first line of defense for *H. pylori* is the gastric mucosal layer, localized in the gastric lamina propria, which consists of gastric epithelial and innate immune cells (dendritic, natural killer, mast cells, etc.). These cells are activated during the pre-infection stage of the *H. pylori* gastric infection, causing a significant rise in the expressions of IL-1, IL-6, and IL-8 in the antrum region and CD4+ and CD8+ T cell recruitment. Immune cells are then prone to secreting cytokines such as interferon γ (IFN-γ) and tumor necrosis factor-α (TNF-α), and interleukins such as IL-1, IL-6, IL-7, IL-8, IL-10, IL-17, and IL-17A [4,5,6]. *H. pylori* penetration into the epithelial mucus layer induces inflammation and produces a phagocytic-rich environment. These phagocytic cells produce their chemical responses, including reactive oxygen species (ROS) and nitric oxide (NO) [7]. All *H. pylori* strains are able to detoxify ROS, producing catalase and superoxide dismutase. Furthermore, *H. pylori* arginase limits NO production by macrophage, neutrophils, and epithelial cells [8,9]. It was demonstrated that non-opsonized *H. pylori* can resist phagocytosis, targeting the NADPH oxidase and disrupting it; this leads to phagosome dysfunction, since superoxide ions diffuse into the extracellular space rather than being located in the phagosome [10]. Finally, the bacterium manages to survive, inducing mitochondria-dependent apoptosis, particularly of macrophages (Figure 1) [11,12].

The recognition of PAMPs is gained through PRRs; *H*. *pylori* has an efficient process to escape those receptors through different camouflages of its molecular pattern. Moreover, PAMPs do not always induce a pro-inflammatory response and/or pathogen clearance but can also dampen immune responses [13]. Here, we summarize some of the most common bacterial PAMPs and how they are modified from *H. pylori* to make their recognition inefficient.

## 2. Lipopolysaccharide

LPS consists of three different domains [14]: the more or less acylated and phosphorylated lipid A of the bacterial outer membrane; the core oligosaccharide (inner and outer) linked by 3-deoxy-d-manno-octulosonic acid (Kdo) to lipid A; and the so-called O-antigen or O-specific polysaccharide, with the latter two facing the aqueous environment. Lipopolysaccharides encompassing all three regions are called smooth (S)-form LPS, while LPS lacking the O-antigen are indicated as rough (R)-form LPS or lipooligosaccharide (LOS).

The *H. pylori* LPS structure plays an essential role in the bacterial immune escape. Compared to other bacterial LPS, such as those of *Escherichia coli*, *H. pylori* LPS has ~1000 fold less endotoxicity [15], and this reduction is attributed to three significant modifications of the lipid A. The first modification is a hypo-acylation pattern; indeed, *H. pylori* Lipid A is tetra-acylated compared to hexa- or penta-acylated chains of other bacteria. Interestingly, *H. pylori* synthesizes a hexa-acylated lipid A that is subsequently deacylated [16]. Second, hypo-acylated fatty acids have longer carbon chain lengths (18-carbon and 16-carbon chains) compared to the optimal chain lengths (12- and 14-carbon chains) required for robust TLR4 activation [17]. Lastly, *H. pylori* LPS is hypo-phosphorylated, since phosphate groups from the 1ʹ- and 4ʹ-positions of the lipid A backbone are removed [18]. This modification reduces the binding with cationic antimicrobial peptides (CAMPs), such as polymyxin B, and escapes detection by TLRs [15,19].

The role of TLR4 in immune activation is controversial; the TLR (or TLRs) involved in the residual detection of *H. pylori* LPS remains a matter of debate. On the one hand, several studies enrolling purified LPS consider TLR4 as a classical LPS sensor [20,21], while on the other hand, further studies suggest that TLR2 is the primary receptor of *H. pylori* LPS [22,23].

A unique feature of the O-chain of the LPS of most *H. pylori* strains is the expression of Lewis (Le) antigens, mimicking the ones expressed by gastric epithelium [24]. This camouflage assists bacteria in escaping from the host’s immune responses and may also induce autoimmune disorders, contributing to the severity and chronicity of infection [25].

In most strains, both Lewis X (LeX) and Lewis Y (LeY) can be found in certain regions of the O-antigen. Some strains also display Lewis A (LeA) and B (LeB) antigens or can have alternative O-antigen structures [26]. *H. pylori* takes advantage of this molecular mimicry, as LeX and LeY interact with the C-type lectin DC-SIGN (Dendritic Cell-Specific Intercellular adhesion molecule-3-Grabbing Non-integrin), leading the immune system to down-regulate the inflammatory response [27].

C-type lectin receptors (CLRs) are members of the PRR family and induce many immune system genes. DC-SIGN is a member of the CLR family and is primarily expressed in DCs. DC-SIGN recognizes predominantly N-linked high-mannose oligosaccharides and branched fucosylated structures and may have two different roles. Fucose residues on *H. pylori* activate the DC-SIGN, stimulating its response. Fucosylated ligand binding can activate NF-kB, inducing T cell maturation and Th1/Th2 polarization [28]. *H. pylori* can also target these receptors to limit cell proliferation and LeX^−^/LeY^−^ strains can escape all these recognition processes [29].

**Figure 2:**
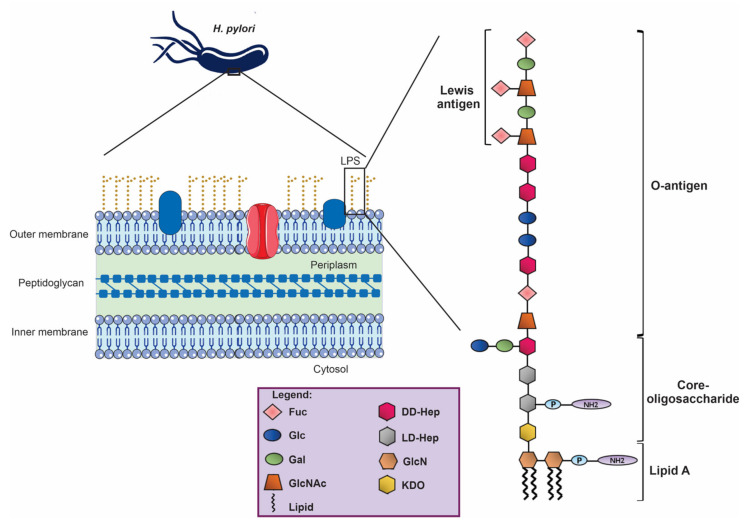
*H. pylori* LPS structure. Lacking the canonical inner and outer core organization, *H. pylori* LPS shows a short core and a longer O-antigen with a terminal Lewis antigen.

Li et al. established the first complete LPS biosynthesis pathway using *H. pylori* strain G27 as a model and redefined the core oligosaccharide domain as a hexasaccharide (Glc-Gal-DD-HepIII-LD-HepII-LD-HepI-KDO), which is decorated with a long O-antigen encompassing the trisaccharide (DD-Hep-Fuc-GlcNAc) termed as Trio; a variable glucan (homopolymer of Glc); a DD-heptane (homopolymer of Hep); and terminal Lewis antigens [30]. *H. pylori* LPS lacks the canonical inner and outer core organization. Instead, it displays a short core and a longer O-antigen encompassing residues previously assigned as the outer core domain (Figure 2).

Analyzing this pathway in a large panel of *H. pylori* strains from different ethnic origins, they found that the genes involved in LPS heptane incorporation are lacking in East Asian strains [31]. Given the high gastric cancer (GC) rate in East Asia, this observation opens exciting questions on a potential role of LPS heptane in *H. pylori* pathogenesis.

## 3. Metabolites

### 3.1. HBP and ADP-Heptose

It has recently been demonstrated that heptose metabolites of LPS inner core biosynthesis can be considered novel PAMPs of Gram-negative bacteria as they activate human epithelial cells at early times of infection via the ALPK1-TIFA axis.

**Figure 3:**
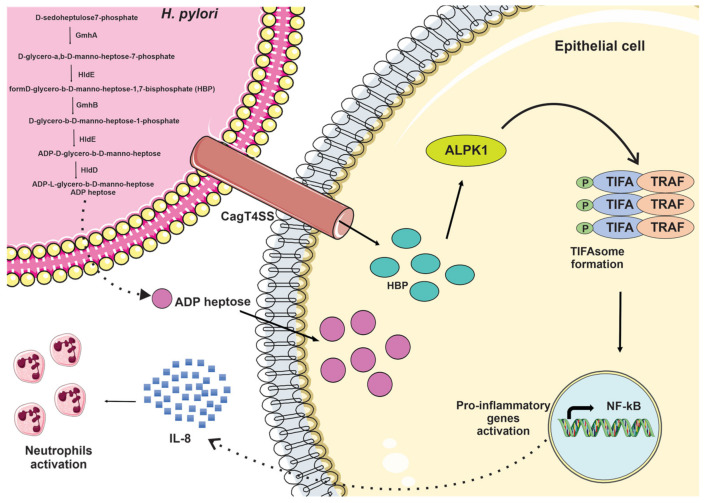
Schematic release and action of *H. pylori* heptose metabolites. HBP and ADP-heptose enter epithelial cells to induce TIFAsome formation and NF-kB activation.

The inner core of LPS typically consists of 3-deoxy-D-manno-octulosonic acid and L,D-heptose units. The precursor of the L,D-heptose units is the ADP heptose (ADP-L-glycero-b-D-manno-heptose), synthesized in a five-step pathway starting from D-sedoheptulose 7-phosphate [32]. The latter is converted into D-glycero-a,b-D-manno-heptose-7-phosphate by the sedoheptulose-7-phosphate isomerase GmhA; phosphorylated to form D-glycero-b-D-manno-heptose-1,7-bisphosphate (HBP) by the bifunctional kinase or adenylyltransferase HldE; then dephosphorylated to D-glycero-b-D-manno-heptose-1-phosphate by the D-glycero-b-D-manno-heptose-1,7-bisphosphate-7-phosphatase (GmhB); followed by nucleotide activation by the formation of ADP-D-glycero-b-D-manno-heptose by HldE; and finally, epimerization to ADP heptose by the epimerase HldD (Figure 3). The resulting activated heptose unit is then integrated into the LPS core region.

The intermediate metabolite HBP was first identified as a potent mediator of NF-kB activation by *Neisseria* species; during the infection, it is released into the cytosol of infected epithelial cells via phagocytosis, activating TRAF-interacting protein with forkhead-associated domain (TIFA) dependent immunity [33].

Subsequently, Stein and co-workers demonstrated that the Cag type-IV secretion system (CagT4SS) is involved in HBP translocation into host cells, inducing the secretion of the pro-inflammatory cytokine IL-8 and activation of NF-kB [34]. Indeed, it was also demonstrated that, in gastric epithelial cells infected with *H. pylori,* NF-kB activation was stimulated by HBP upon transfection, presumably mimicking HBP translocation to the cytosol via the CagT4SS with the involvement of Alpha-kinase 1 (ALPK1) and TIFA [35].

Pfannuck and co-workers recently found that a derivative of HBP, ADP-heptose, is produced by *H. pylori* at concentrations more than 10 times higher than HBP. At the same time, it is far more potent, activating ALPK1-TIFA-controlled innate immune responses in epithelial cells at 100-fold lower concentrations [36]. Furthermore, ADP heptose was demonstrated to be a ligand of ALPK1, suggesting that HBP is converted inside the host cell to ADP-heptose-7-phosphate, which can also activate ALPK1, but to a lesser extent [37].

ALPK1 belongs to the family of alpha kinases. These serine/threonine kinases display no homology with conventional kinases in their catalytic domains and are implicated in the apical protein transport to maintain polarity in epithelial cells [38]. ALPK1 was then identified as a PRR in infections by Gram-negative bacteria, since its catalytic activity is essential for the self-oligomerization of TIFA [35,39]. The latter was first described as a TRAF2-binding protein for its ability to activate NF-kB and AP-1 [40]. Through the involvement of the FHA domain and the phosphorylation of threonine at position 9 [41], TIFA undergoes self-oligomerization, thus promoting the oligomerization and ubiquitinylation of TRAF6, leading to the activation of the IKK complex (Figure 3) [42].

The classical activation of NF-kB depends on the formation of oligomeric TIFA-TRAF6 complexes [39], and TAK1 (TGFβ-activated kinase 1) binding to TRAF6 upon *H. pylori* infection seems to be dependent on TIFA. At the same time, TRAF3, TRAF2, and cIAP1 (cellular inhibitor of apoptosis 1) are probably involved in the formation of TIFAsomes. Therefore, *H. pylori* infection can induce different TIFAsome complexes that activate the classical and alternative NF-kB pathways [43]. This dual possibility could trigger various cellular events, leading to the induction of inflammatory cytokines by the classical NF-kB pathway and more complex physiological changes due to the alternative NF-kB pathway that can lead to gastric pathologies [44].

Hence, *H. pylori* heptose metabolites play a significant role in early phagocytic cell activation, including NF-kB activation and IL-8 production. Bacterial purified lysates and pure ADP-heptose on its own, in the absence of other bacterial PAMPs, are able to strongly activate Thp-1 cells and human primary monocytes/macrophages, differentiating monocytes into macrophages of predominantly M1 type, and this activation was significantly reduced upon TIFA knock-down. In this cell model, the CagT4SS was less necessary than in epithelial cells. In contrast, active heptose biosynthesis or pure ADP-heptose was required and sufficient for an early innate response and NF-kB activation [45].

### 3.2. Cholesterol Derivatives

*H. pylori* produces different lipids, mainly various cholesterol metabolites [46]. Indeed, *H. pylori* extracts cholesterol from the host and converts it to specific cholesteryl glucosides, such as cholesteryl acyl α-glucosides (αCAGs) [47,48]. One of the hypothesized roles of these cholesterol metabolites is to suppress host immunity [49], although their immunomodulatory properties and host pattern recognition receptors have not yet been determined.

Recently, αCAG and cholesteryl phosphatidyl α-glucoside (αCPG) were identified as noncanonical ligands for Mincle (Clec4e) and DCAR (Clec4b1). During chronic infection, *H. pylori*-specific T cell responses and gastritis were ameliorated in Mincle-deficient mice, although bacterial burdens remained unchanged. Furthermore, a mutant *H. pylori* strain lacking αCAG and αCPG exhibited an impaired ability to cause gastritis. Thus, *H. pylori*-specific modification of host cholesterol plays a pathophysiological role that exacerbates gastric inflammation by triggering C-type lectin receptors [50].

## 4. Bacterial Proteins and Peptides

### 4.1. Flagellin

The natural ligand of TLR5 is flagellin, specifically the highly conserved N-terminus of the D1 domain [51]. Since *H. pylori* is a flagellated bacterium, TLR5, expressed in the gastric epithelium, should be able to recognize this pathogen and to mediate the pro-inflammatory signaling cascades. However, *H. pylori* flagellin is not recognized by TLR5 [52] due to a mutation in the conserved domain of FlaA. This mutation, which occurs in the D0-D1 domain between amino acids 89 and 96, masks *H. pylori* flagella from TLR5 binding and prevents its activation. The importance of these residues was demonstrated by Andersen-Nissen and co-workers, who substituted them into the corresponding region of the *Salmonella enterica* serovar Typhimurium FliC, making this flagellin unable to activate TLR5 similar to that of *H. pylori* [53]. The low intrinsic activity of its mutated FlaA confers to *H. pylori* the opportunity to avoid permanent TLR5 activation. These data suggest an essential role for *H. pylori* FlaA in maintaining persistence within the gastric niche by dampening the innate immune response. Bacterium persistence is also due to the formation of *H. pylori* aggregates in the mucus layer, which do not affect *flaA* gene transcription [54].

Indeed, *H. pylori* evolved a CagT4SS-dependent but flagellin-independent ability to activate TLR5. CagY and CagL, exposed at the tip of the secretion system, can directly bind to TLR5. Since the T4SS-pilus is induced upon host cell contact, the TLR5 activation can be turned on and off by exposing or hiding CagY and CagL, deregulating or hyper-regulating host immunity [55,56].

Recently, new polymorphisms of TLR5 have been associated with a high risk for gastric carcinoma consequent to *H. pylori* infection, likely due to an acquired ability to recognize *H. pylori* flagellin [57].

### 4.2. Hp(2-20) Peptide

Formyl Peptide Receptors (FPRs), comprising FPR1, FPR2, and FPR3, can be considered another class of PRRs. They are highly expressed by innate immune cells but are also presented by epithelial cells of the mucosal systems, including the gastrointestinal tract [58]. FPRs were discovered as receptors with a high affinity for N-formylated peptides, either derived from bacterial signal peptides or released from mitochondria of damaged host cells. Indeed, these receptors interact with several different ligands other than the N-formyl-methionine-leucyl-phenylalanine (fMLF), the prototype of N-formylated peptides [59].

Hp(2-20) is a non-formylated, cecropin-like peptide released during *H. pylori* growth with a high affinity for formyl peptide receptor like-1 (FPR2). Hp(2-20) shows different functional features. When *H. pylori* colonizes the human stomach, Hp(2-20) contributes to activating the innate immune response by interacting with the FPR2 expressed on gastric epithelial cells. Indeed, this interaction favors gastric mucosa inflammation by initiating a cell signaling cascade, which leads to cytokine release, recruitment of inflammatory cells, and stimulation of NADPH oxidase-dependent superoxide generation [60]. Hp(2-20) also stimulates gastric epithelial cell migration and proliferation, inducing the expression of vascular endothelial growth factor (VEGF), which aids in repairing the gastric mucosa after induced injury [61]. These events favor the host defense and the restoration of homeostasis. On the other hand, Hp(2-20) is also an antimicrobial peptide (AMP), which plays an essential role in *H. pylori* gastric mucosa persistence by acting as a bactericidal molecule, thus conferring an advantage over other microorganisms [62].

## 5. Extracellular DNA and RNA

During microbial infection, DNA can be actively secreted or released due to degradation from invading microbes or damaged host cells. TLR9, an endosome-bound transmembrane receptor, detects these aberrant DNAs and promotes an immune response [63]. TLR9 was initially reported to recognize pathogenic DNAs based upon the presence of hypo-methylated CpG DNA motifs; however, accumulating evidence suggests that TLR9 can also detect DNA in a sequence-independent manner by recognition of the saccharide backbone [64,65]. Since TLR9 is constrained to the endosome, cells of the immune system must internalize pathogens or pathogenic DNA before TLR9 can detect it. Most immune cells accomplish this task through receptor-mediated endocytosis in response to scavenger receptor binding, phagocytosis of complement-mediated opsonized material, or Fc receptor-mediated uptake of antibody opsonized material or through a combination of these processes [66].

Regarding the gastric compartment, the expression of this receptor differs between healthy individuals and individuals who are infected with *H. pylori*. TLR9 is located in the apical compartment of the gastric epithelial cells in healthy controls and in the basolateral compartment in individuals with infections [67]. Additionally, it seems mandatory for TLR9 activation that *H. pylori* translocates its DNA into the cells by cancer-associated CagT4SS, allowing for its engagement by the receptor [68].

In the context of dendritic cells, the scenario changes, as the intracellular delivery of *H. pylori* DNA by lipofection efficiently activates endosomal localized TLR9, promoting an anti-inflammatory rather than a pro-inflammatory response [69]. In the same way, in the early stages of a mouse model infection, TLR9 activation induced an anti-inflammatory cascade [13]. *H. pylori* colonization is inversely correlated with the risk of developing inflammatory bowel diseases (IBD), and this seems to be caused by a specific immunoregulatory sequence (TTTAGGG) unique to the *H. pylori* genome. Therefore, *H. pylori* DNA can be used therapeutically to treat experimentally induced IBD in mice [69,70].

Therefore, the hypothetical scenario is that *H. pylori* may utilize TLR9 signaling to dampen the inflammatory response during the acute phase to establish infection; however, at a certain point, when cells have lost their polarity due to inflammatory micro-environment, TLR9 may increase pro-inflammatory cascades and further favor the progression toward gastric cancer.

While *H. pylori* DNA is sensed by TLR9, the detection of the bacterial RNA in dendritic cells seems to be mediated by endosomal localized TLR8 and by a cytoplasmic nucleic acid sensor, RIG-I, which belongs to the RIG-like helicase receptor family (RLR). RIG-I is able to recognize 5ʹ triphosphorylated *H. pylori* RNA and to promote the following IRF3- and IRF7-dependent induction of type I interferons (IFN α and β) by dendritic cells. It is not yet clear whether *H. pylori*–RNA sensing has mainly pro-inflammatory or anti-inflammatory effects [71].

## 6. Outer Membrane Vesicles

Gram-negative cell surfaces constantly release outer membrane vesicles (OMVs), which contain different components with various functions: increasing virulence, mediating bacterial cell–cell communication, modulating host immune response, and more.

The vesicle diameter ranges between 10 and 300 nm, consisting of lipids, proteins, LPS, phospholipids, DNA, RNA, proteins, inner membrane (IM), periplasm, outer membrane (OM), and other molecules. Several studies have provided that OMVs have opposite effects, both harmful and defensive [72].

Since they deliver many pathogenic bacterial proteins, they induce pro-inflammatory responses in the host. Indeed, OMVs from the Gram-negative pathogens *H. pylori*, *Neisseria*, *Pseudomonas*, *Campylobacter*, and *Vibrio* promote the secretion of interleukin-8 (IL-8) by non-phagocytic epithelial cells [73].

*H. pylori* takes advantage of OMVs to cross the cellular barrier and efficiently translocate antigens through the gastric mucosa up to immune cells in tissues or even to circulation. Blebbing of *H. pylori* OM was detected in bacteria cultures during the late stationary growth phase and biofilm formation and in gastric biopsies of individuals with infection [74,75].

Regarding their content, Olofsson and co-workers showed that vesicles of this bacterium contain OM but not IM molecules [76]. The content of OMVs might vary among *H. pylori* strains. *H. pylori* OMVs contain different phospholipids: phosphatidylglycerol (PG); phosphatidylethanolamine (PE); lyso PE (LPE); phosphatidylcholine (PC); lyso PC (LPC); and cardiolipin [76]. LPS is another critical component of *H. pylori* OMVs; in particular, the O-chain of *H. pylori* LPS displays LewisXY antigens in iron-reach conditions, whereas the presence of LewisY antigens is reduced in iron-limited conditions [77].

Recent proteomic analyses of small and large OMVs revealed that smaller OMVs contained significantly fewer proteins than larger ones. Moreover, larger *H. pylori* OMVs contain bacterial adhesion proteins absent from smaller OMVs, which may facilitate their entry into host cells via receptor-mediated endocytosis. Twenty-four proteins were found to be common to both small and large OMVs; these were mainly associated with virulence, including vacuolating cytotoxin (VacA); CagA; blood group antigen-binding adhesin (BabA); sialic acid-binding adhesin (SabA); outer inflammatory protein A (OipA); *H. pylori* neutrophil-activating protein (HP-NAP); adherence associated lipoprotein (AlpA); urease; and catalase (KatA) [9,78].

Regarding internalization mechanism, *H. pylori* OMVs can enter epithelial cells via macropinocytosis, clathrin, and caveolin-dependent endocytosis. Indeed, smaller OMVs with sizes ranging from 20 to 100 nm preferentially use caveolin-mediated endocytosis. In contrast, larger OMVs ranging between 90 and 450 nm use macropinocytosis and endocytosis [78].

One of the first PRRs found to become activated on *H. pylori* infection was NOD1, able to detect *H. pylori* peptidoglycan. Initially, it seemed that peptidoglycan could be delivered into the cytoplasm of host epithelial cells only by *H. pylori* strains with a functional CagT4SS [79]. It is now clear that peptidoglycan can also be delivered to NOD1 from outer-membrane vesicles (OMVs) of Cag PAI-negative strains of *H. pylori*. Intragastric delivery of OMVs in mice induces innate and adaptive immune responses through a NOD1-dependent but TLR-independent mechanism [80]. The delivery of peptidoglycan by both OMVs and the T4SS occurs at cholesterol-rich lipid rafts [80,81].

NOD1 activates NF-kB signaling pathways and the transcription factor AP1 via ERK- and p38-dependent pathways [82]. A direct effect of NOD1 signaling activation in gastric epithelial cells is the production of the antimicrobial peptide β-defensin 2, with consequent *H. pylori* killing [83]. It has recently been described an alternative NOD1-dependent signaling pathway, which activates the IRF3 and IRF7 transcription factors suggested to induce type I IFNs essential for *H. pylori*-specific inflammatory response [84].

Hence, *H. pylori* OMVs can activate NF-kB, MAPK, and ERK pathways, thus modulating the expression of genes involved in the inflammatory response, proliferation, and carcinogenesis [85]. Moreover, it was shown that *H. pylori* OMVs can induce IL-8 production by gastric epithelial cells independently of VacA [86].

This pro-inflammatory potential suggests that they can participate in the carcinogenesis process. This hypothesis was assessed by the observation that *H. pylori* OMVs induce inflammation in a mice model of gastric cancer and that their presence is significantly higher in the gastric juices of gastric cancer patients than in normal controls [87].

AGS human gastric epithelial cells treated with *H. pylori* OMVs showed increased micronuclei formation, alteration in iron metabolism, and glutathione loss [88].

However, some *H. pylori* OMV components, such as LPS, may stimulate opposite outcomes. The endotoxic activity of *H. pylori* LPS is lower than LPS from other bacteria due to the different structure of lipid A, as already discussed. OMVs decorated with *H. pylori* LPS bearing Lewis antigens in O-chains may chronically stimulate the host immune cells to produce autoantibodies, playing an essential role in autoimmune processes related to *H. pylori* infection [89].

## 7. Conclusions

*H. pylori*, like all Gram-negative bacteria, stimulates the innate immune response at the onset of the infection by PAMP signals detected by host PRRs. Many of these patterns (LPS, flagellin) have been extensively analyzed in *Helicobacter pylori* and have shown structural features that differentiate them from other bacteria’s more efficient systems. These mutated *H. pylori* PAMPs are less recognized by the host receptors, contributing to the establishment of a silent and persistent infection. Some others (ADP-heptose, nucleic acids, OMVs) are not yet fully explored and deserve a more detailed characterization in order to definitely assess their role during the infection: host friends or foes?

## Data Availability

Not applicable.
